# Immunomodulatory Effects of MSC-Derived ExtracellularVesicles in Celiac Disease

**DOI:** 10.3390/ijms27209249

**Published:** 2026-10-18

**Authors:** Rachele Ciccocioppo, Ilaria Mottola, Valeria Zuliani, Annalisa Adamo, Serena Vitale, Mauro Krampera, Alessandra Camarca, Vera Rotondi Aufiero, Laura Pisapia, Fabiana Capuano, Giuseppe Mazzarella, Giovanna Del Pozzo, Carmen Gianfrani

**Affiliations:** 1 Gastroenterology & Endoscopy Unit, Department of Medicine and Aging Sciences, Ospedale Clinicizzato SS. Annunziata, Gabriele d’Annunzio University, 66100 Chieti, Italy; rachele.ciccocioppo@unich.it; 2 Institute of Biochemistry and Cell Biology, CNR, 80131 Naples, Italycarmen.gianfrani@ibbc.cnr.it (C.G.); 3 European Laboratory for the Investigation of Food Induced Diseases (ELFID), 80131 Naples, Italy; giuseppe.mazzarella@cnr.it; 4 Haematology Unit, Department of Medicine, A.O.U.I. Policlinico G.B. Rossi, University of Verona, 37134 Verona, Italy; 5 Hematology and Bone Marrow Transplant Unit, Section of Biomedicine of Innovation, Department of Engineering for Innovative Medicine, University of Verona, 37134 Verona, Italy; 6 Institute of Food Sciences, CNR, 83100 Avellino, Italy; alessandra.camarca@cnr.it; 7 Institute of Genetics and Biophysics, CNR, 80131 Naples, Italygiovanna.delpozzo@igb.cnr.it (G.D.P.); 8 Gastroenterology Unit, San G. Moscati Hospital, 83100 Avellino, Italy

**Keywords:** coeliac disease, extracellular vesicles, immunomodulation, mesenchymal stromal cells, gluten protein, mucosal inflammation

## Abstract

Coeliac disease (CeD) is the most frequent chronic enteropathy, in which intestinal lesions are caused by a dysregulated immune response to gluten proteins. We aimed to investigate whether extracellular vesicles (EVs) from mesenchymal stem cells (MSC) exert immunomodulatory effects in experimental models of CeD and are potentially useful to treat CeD. A population of MSCs was obtained from bone marrow blood of adult healthy donors. EVs were purified by ultracentrifugation of MSC-supernatants and characterized in terms of size, protein content and expression of surface markers. The immunomodulatory effects of MSC-EVs were evaluated in vitro on gliadin-specific T-cell lines by detection of interferon (IFN)-γ production and ex vivo by using duodenal mucosa biopsies by measuring lymphocyte infiltration (CD3 and CD25 positive cells), IFN-γ and interleukin (IL)-10 secretion. Moreover, the MSC-EVs effects on B-cells in terms of HLA-DQ/DR and CD83/CD86 expression upon antigen stimulation was also evaluated. EVs obtained from non-celiac adult fibroblasts were used as controls. A significant and dose-dependent reduction of IFN-γ production by T cells in response to gliadin stimulation was observed in the presence of MSC-EVs (*p* < 0.05), but not with fibroblast-EVs. No modification of the expression of HLA class II molecules and costimulatory CD83 and CD86 markers on B cells upon EV-MSC incubation was found at any tested dose. Moreover, EVs from both MSCs and fibroblasts inhibited the gliadin-induced expansion of CD3+ and CD25+ T cells in organ cultures (*p* < 0.05), while increasing the IL-10 production (*p* < 0.05) and reducing IFN-γ (*p* = n.s) in organ supernatants. MSC- EVs modulated selected components of the immune response to gliadin in experimental models of CeD, including antigen-induced IFN-γ production and cellular responses in intestinal organ cultures. The effects observed in organ cultures were not exclusive to MSC-EVs, as EVs from fibroblast also modulated cellular responses. These findings provide a rationale for further preclinical investigation of EV-mediated immunomodulation in CeD.

## 1. Introduction

Celiac disease (CeD) is an immune-mediated enteropathy triggered and sustained by the ingestion of gluten proteins in genetically susceptible individuals, and its treatment currently consists of a strict and lifelong gluten-free diet (GFD) [1]. CeD affects approximately 1% of the general population worldwide, although a substantial proportion of affected individuals remains undiagnosed [1,2]. Beyond intestinal manifestations, CeD may be associated with extraintestinal symptoms and nutritional and systemic complications, contributing to a relevant long-term health burden [2,3]. Although a strict GFD generally leads to clinical improvement and intestinal mucosal recovery, maintaining lifelong dietary restriction can substantially affect patients’ quality of life and nutritional status and represents a significant long-term burden [2,3].

These limitations have prompted the investigation of several pharmacological and biological strategies aimed at complementing or, in selected circumstances, potentially replacing the GFD. Several candidate approaches are currently being evaluated at proof-of-concept, preclinical, or clinical stages [4]. However, the complexity of CeD pathogenesis, the absence of a widely accepted animal model that fully reproduces the human disease [5], and the need to establish both efficacy and safety have hampered the development of alternative therapeutic approaches. Thus, the identification of safe and effective strategies capable of modulating the gluten-induced intestinal immune response remains an unmet need in CeD.

In this context, mesenchymal stem cells (MSCs), owing to their immunomodulatory and regenerative properties, have attracted considerable interest in chronic immune-mediated and inflammatory conditions [6]. MSC-based approaches have also been investigated in intestinal disorders characterized by immune dysregulation and mucosal injury, including acute graft-versus-host disease [7], autoimmune enteropathy [8], and refractory CeD [9]. Nonetheless, the clinical use of intact MSCs presents several limitations, including variability in cell survival and function after administration, limited ability to reach and persist at the desired site of action, and challenges related to cell manufacturing, storage, and delivery [10].

Increasing evidence suggests that many of the immunomodulatory and regenerative effects attributed to MSCs are mediated, at least in part, by extracellular vesicles (EVs), nanoscale membrane-enclosed particles carrying bioactive proteins, lipids, and nucleic acids capable of influencing target-cell functions, including immune responses and tissue repair [11,12,13]. This possibility opens novel therapeutic perspectives in immune-mediated disorders aimed at developing cell-free treatment based on the use of MSC-released EVs [14]. On this basis, we hypothesized an immunosuppressive role and investigated the anti-inflammatory effects of EVs obtained from human bone marrow-derived MSCs (MSC-EVs) on well-established CeD experimental in vitro models commonly applied to explore the gluten immune reactivity, i.e., intestinal T-cell lines (TCLs) that are highly reactive to gluten and organ cultures of gut mucosa from CeD patients [15,16].

## 2. Results

### 2.1. MSC-EVs Dampen IFN-γ Secretion by Gliadin-Reactive T-Cells

To assess the immunomodulatory effects of MSC-EVs on gliadin-reactive T cells, we took advantage of short-term TCLs generated from intestinal biopsies of patients with CeD, which represent a sensitive and validated model of T-cell reactivity to gluten [15,17,18]. EVs were isolated from resting and IFN-γ-primed MSC cultures obtained from healthy donors according to a previously published protocol [19] (Supplementary Figure S1A). NTA was used to characterize EV size (Supplementary Figure S1B), while a multiplex bead-based assay confirmed the expression of the EV-associated markers CD63, CD81, and CD9. MSC-derived EVs also expressed CD29, CD105, SSEA-4, and CD146, while lacking hematopoietic, epithelial, and cancer stem cell markers (Supplementary Figure S1C).

**Figure 1:**
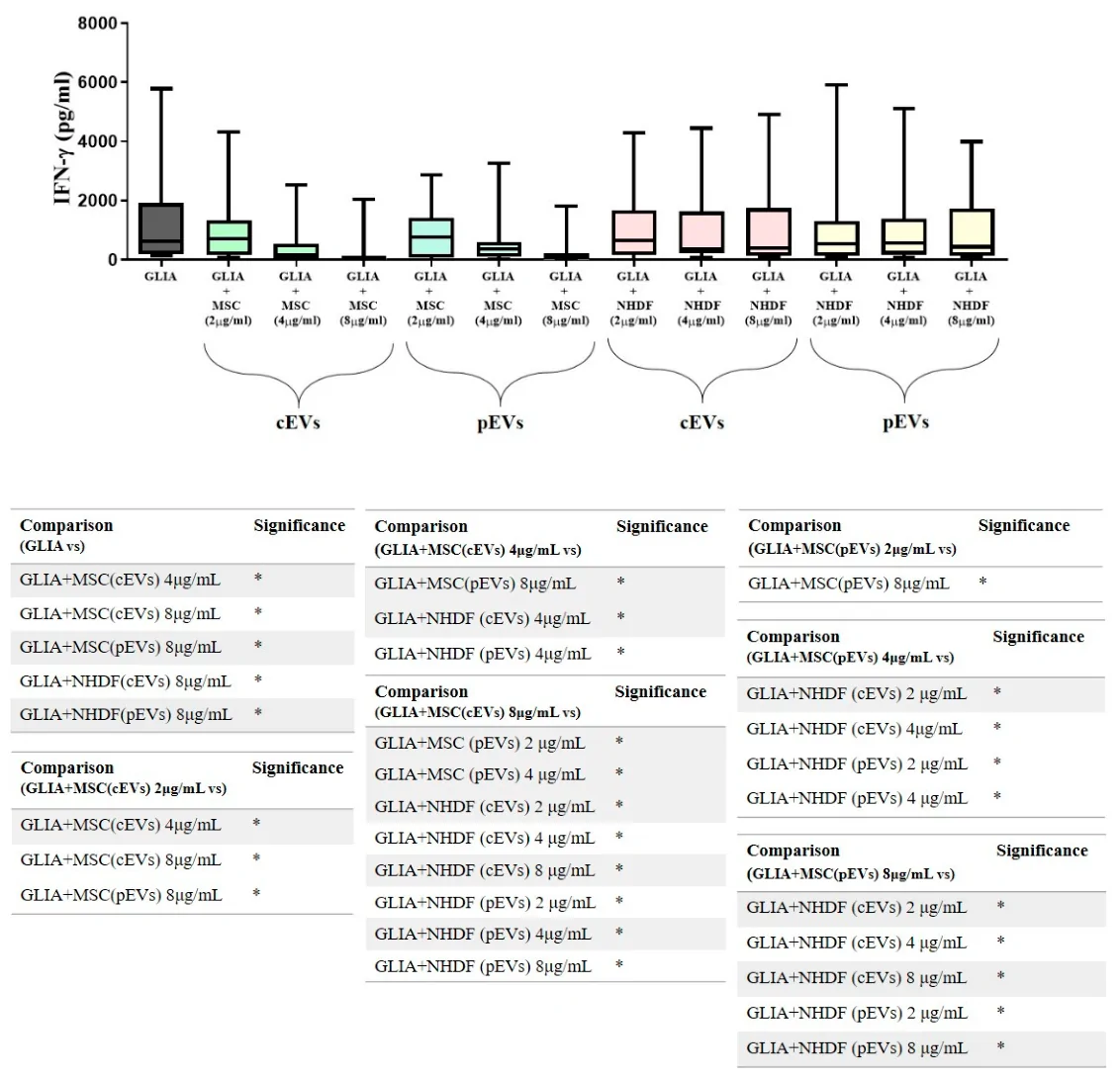
Mesenchymal stem cell-derived extracellular vesicles (MSC-EVs) induce a significant, dose-dependent decrease in interferon-γ secretion in gliadin-responsive short-term T-cell lines. T cell lines generated from celiac gut mucosa were stimulated with gliadin (GLIA) in the presence of EVs isolated from healthy donor bone marrow MSCs or NHDFs, used as control samples. EVs were obtained either from unstimulated (cEVs) or IFN-γ-primed (pEVs) MSCs/NHDFs. T cell activation was evaluated by detection in cell supernatants of IFN-γ production by ELISA. The data (pg/mL) are shown as median (midline), 25th to 75th percentiles (box) and extremes (whiskers). Each experiment was performed in technical duplicate. All statistically significant pairwise comparisons are summarized in the accompanying table, which reports comparisons of GLIA alone with each indicated MSC- or NHDF-derived EV condition, including cEVs and pEVs at the different concentrations tested. The corresponding *p*-values are indicated by asterisks: * *p* < 0.05.

TCLs were stimulated in vitro with gliadin in the presence of MSC-EVs. A significant and dose-dependent reduction in IFN-γ secretion was observed compared with gliadin stimulation alone (Figure 1).

This effect was observed at all tested concentrations of MSC-EVs, even at the lowest dose of 2 μg/mL for both unprimed MSC-EVs (MSC-cEVs; median IFN-γ release: 609.1 pg/mL, range: 70.6–4319) and primed MSC-EVs (MSC-pEVs; median IFN-γ release: 748.1 pg/mL, range: 0–2855.7), and reached a statistical significance at concentrations of both 4 μg/mL (MSC-cEVs: median IFN-γ release 157.2 pg/mL, range 36.8–2531.6; MSC-pEVs: median IFN-γ release 363.2 pg/mL, range 13.4–3271.7; *p* < 0.05 for both) and 8 μg/mL (MSC-cEVs: median IFN-γ release 31.2 pg/mL, range 0–2028.1; MSC-pEVs: median IFN-γ release 120.7 pg/mL, range 0–1809.0; *p* < 0.01 and *p* < 0.05, respectively). Moreover, the inhibitory effect was specific to MSC-EVs, as no changes were observed when using both NHDF-cEVs and NHDF-pEVs.

### 2.2. MSC-EV Incubation Does Not Alter HLA Class II and Costimulatory Molecule Expression on B Cells Presenting Gliadin Peptides

**Figure 2:**
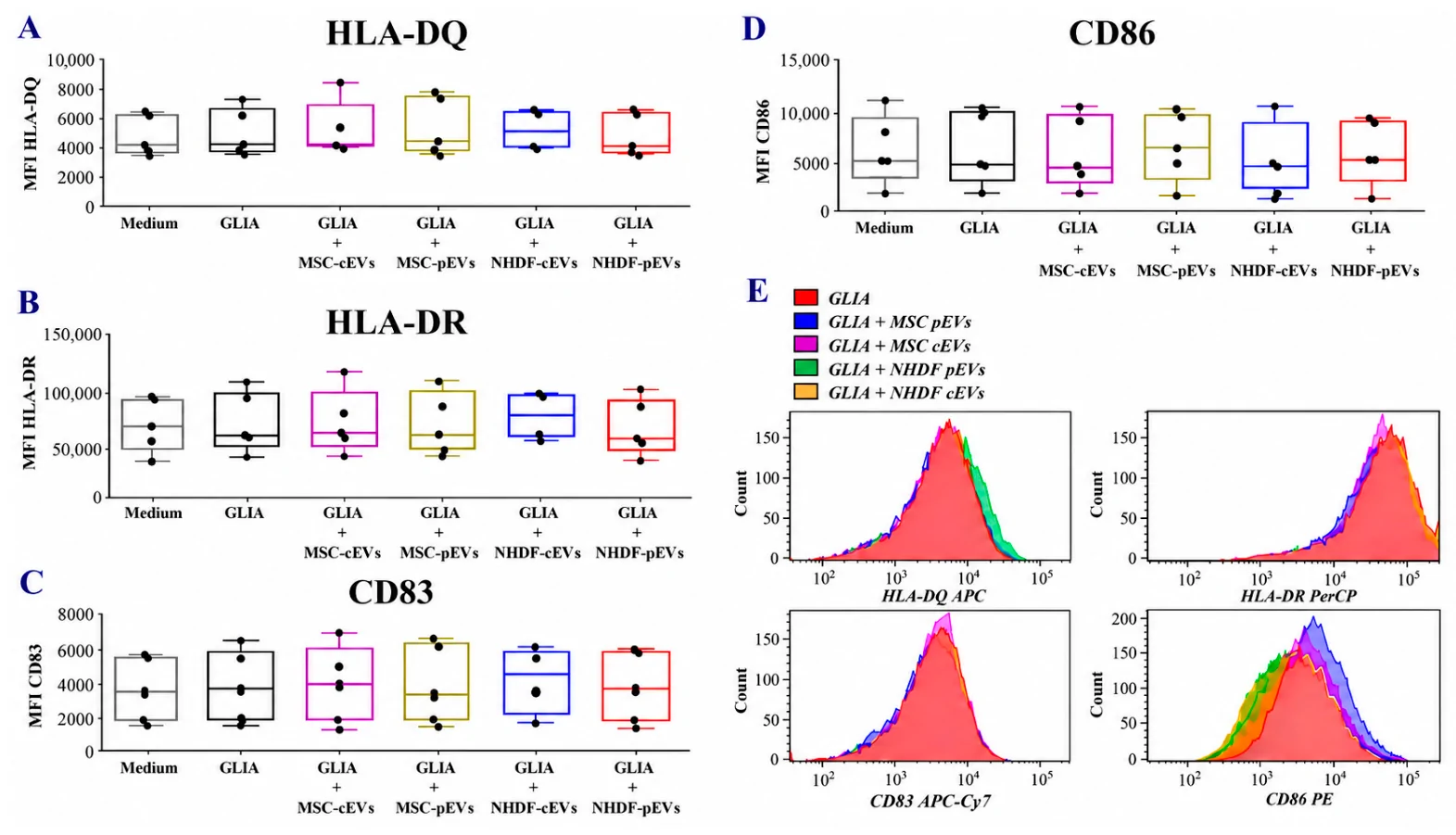
Lack of any effect of extracellular vesicles on the immunophenotype of B-lymphoblastoid cell lines pulsed with gliadin. The graph shows the MFI of cell surface expression of: (**A**) HLA-DQ; (**B**) HLA-DR; (**C**) CD83; (**D**) CD86 measured on B-lymphoblastoid cell lines incubated with different EVs preparations (MSC-cEVs, MSC-pEVs, NHDF-cEVs and NHDF-pEVs) in the presence of gliadin (GLIA). After 48 h of incubation, cells were stained with monoclonal antibody and analyzed by flow cytometry (see the Section 4). Each dot represents a single patient, and mean fluorescence intensity (MFI) results are shown as median (midline), 25th to 75th percentiles (box) and extremes (whiskers). (**E**) Representative flow cytometry graphs for each surface marker.

The presentation of gliadin peptides by HLA-DQ2.5-positive antigen-presenting cells to CD4+ T cells is a pivotal pathogenic step in CeD [15,20]. We therefore investigated whether the inhibitory effect exerted by MSC-EVs on short-term TCLs could be associated with changes in the surface phenotype of B cells involved in gliadin presentation. Immortalized B cells established from five HLA-DQ2.5-positive CeD patients were incubated with gliadin and EV preparations at the highest concentration tested (8 µg/mL). As shown in Figure 2, treatment with EVs derived from either MSCs or fibroblasts, in both primed and unprimed conditions, did not induce significant changes in the surface expression of HLA class II molecules DQ and DR or of the co-stimulatory molecules CD83 and CD86 compared with untreated cells. Notwithstanding these findings, an EV-mediated immunomodulatory effect on the antigen-presentation function of B cells cannot be excluded.

### 2.3. EVs Modulate Cellular and Cytokine Responses in CeD Organ Cultures

**Figure 3:**
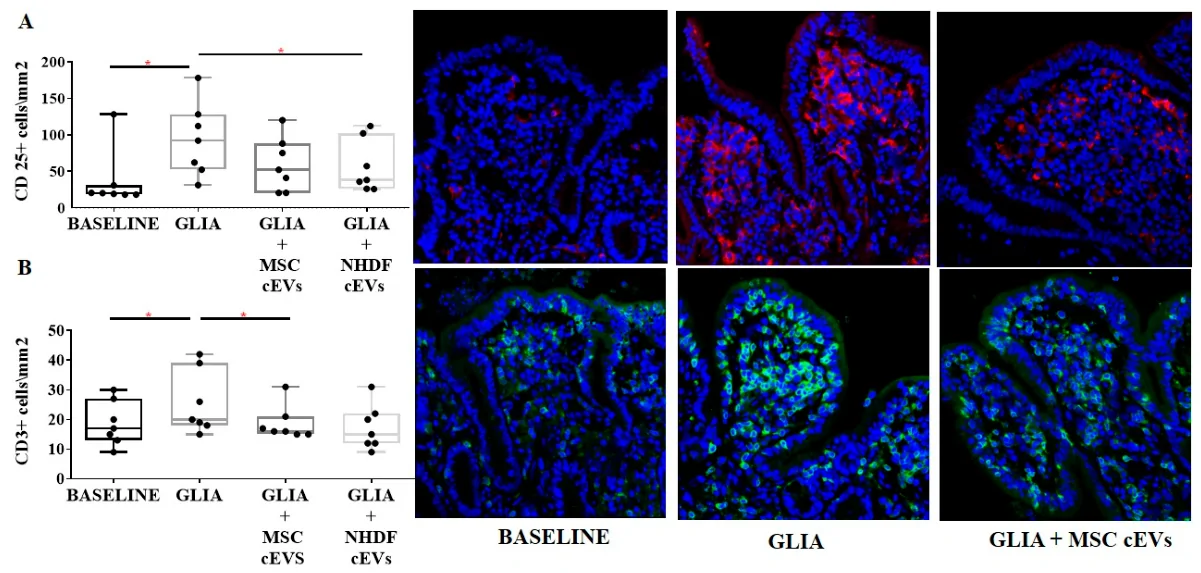
EVs reduces activated lamina propria mononuclear cells and T IELs in organ cultured duodenal biopsies. (**A**) Number of activated CD25+ lamina propria mononuclear cells with only medium (BASELINE), upon gliadin (GLIA) stimulation alone and plus extracellular vesicle (cEVs) batches from both mesenchymal stem cell (MSC) and fibroblast (NHDF) populations. (**B**) Number of CD3+ IELs at BASELINE, upon GLIA stimulation alone and plus MSC-cEVs and NHDF-cEVs, respectively. In the left panels, each dot represents an independent patient-derived biological replicate (*n* = 7). For each patient, the indicated experimental conditions were tested using the same biopsy-derived organ-culture system and are shown as median (midline), 25th to 75th percentiles (box) and extremes (whiskers). * *p* < 0.05. In the right panels, mucosal sections from a representative coeliac patient cultured in vitro at BASELINE, with GLIA alone or in combination with MSC-cEVs, are reported. Activated CD25+ cells are shown in red, CD3+ IELs are shown in green and nuclei are counterstained with DAPI (blue). Original magnification: ×400. A representative fluorescence image from the NHDF-cEV group is provided in the Supplementary Materials (Supplementary Figure S3).

Consistent with previous results from our group [16,17,21] gliadin induced a cell immune activation in the lamina propria and epithelium of small intestinal biopsies from CeD patients, as assessed by CD25+ and CD3+ cell counts in the organ culture system. More in depth, the number of CD25^+^ cells/mm^2^ was significantly increased after stimulation with gliadin as compared to medium alone (median: 92.1, range 31–178 versus 20, range: 18–128; *p* < 0.05; Figure 3A). A similarly decreased number of CD25+ cells/mm^2^ was observed in biopsies incubated with gliadin plus MSC-cEVs (median: 52, range: 20–120; *p* = 0.43) and NHDF-cEVs (median: 36, range: 26–112; *p* < 0.05) compared to samples cultured with gliadin alone (Figure 3A), although statistical significance was reached only for NHDF-cEVs. As far as the CD3+ cell count is regarded, the antigen induced a significant increase (*p* < 0.05) of IELs in comparison to medium alone (median: 20, range: 15–42 versus 17, range: 15–30; Figure 3B).

By contrast, a statistically significant decrease of the number of CD3+ cells was observed in biopsies incubated with gliadin plus MSC-cEVs (median: 16, range: 15–31, *p* < 0.05 vs. gliadin alone) compared to those cultured with gliadin alone, and a similar, although not significant reduction, was observed with NHDF-cEVs (median: 15, range: 9–31, *p* = 0.078 vs. gliadin alone).

### 2.4. Reduction in IFN-γ/IL-10 Ratio in MSC-EV Cultured Biopsies

Since exposure of celiac gut mucosa to gliadin is known to induce the expression not only of inflammatory cytokines, but also of regulatory mediators that contribute to counterbalancing inflammation [22,23], we next evaluated the release of both IFN-γ and IL-10 in the supernatants of organ cultures.

**Figure 4:**
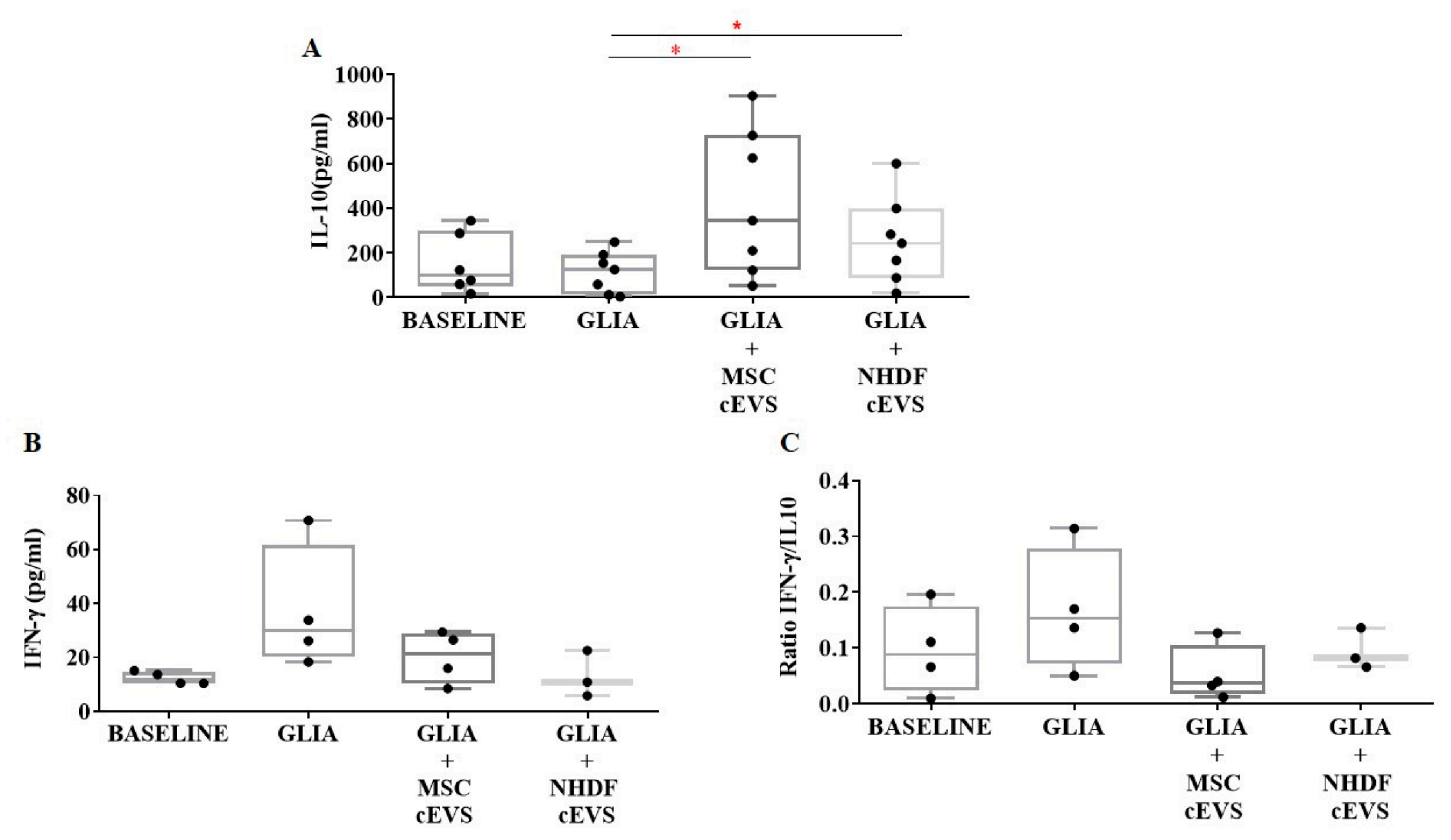
EVs affect the cytokine profile in organ culture supernatants. (**A**) Levels of IL-10 and (**B**) Levels of IFN-γ, released by CeD intestinal biopsies after 24-h of organ culture (*n* = 7 and *n* = 4, respectively) as measured by sandwich ELISA. (**C**) The ratio between concentration values for both IFN-γ and IL-10 was calculated for each experimental point. Each dot represents a single patient (mean value of technical triplicate), and results are shown as median (midline), 25th to 75th percentiles (box) and extremes (whiskers) * *p* < 0.05.

As shown in Figure 4A, the addition of both EV preparations enhanced IL-10 secretion compared with medium alone and gliadin-stimulated cultures. In fact, increased IL-10 levels were observed in the presence of MSC-cEVs (median: 276.9 pg/mL, range 52.2–726) and of NHDF-cEVs (median: 204.3 pg/mL, range 19.5–600), relative to medium alone (median: 100 pg/mL, range 17–344) and gliadin-stimulated cultures (median: 91.9 pg/mL, range 3.7–248.4). Notably, the increase in IL-10 secretion was statistically significant for both EVs preparations when compared with gliadin-stimulated cultures (*p* < 0.05). These findings are consistent with a possible modulation of regulatory responses by EVs, although the contribution of MSC-EVs cannot be distinguished from that of NHDF-EVs in this organ-culture model. In parallel (Figure 4B) we assessed the IFN-γ production in a subgroup of organ culture samples and observed a reduction in IFN-γ secretion when cultures were stimulated with gliadin in the presence of EVs. Specifically, gliadin stimulation was associated with an increase in IFN-γ production compared with baseline; however, this difference did not reach statistical significance (*p* = 0.125). (IFN-γ amount, median: 30 pg/mL, range 18.3–70.7 and 12.1, range 10.4–13.7 in gliadin-stimulated and baseline cultures, respectively, *p* = 0.125). The addition of the EVs derived from either MSC or NHDF reduced IFN-γ levels (median: 21.2 pg/mL; range 8.5–29.4 and 10.9, range 5.8–22.6 in MSC-cEVs and NHDF-cEVs, treated cultures respectively. Although these differences did not reach statistical significance, the observed pattern was consistent with a possible attenuation of IFN-γ production in the presence of EVs. Given the small number of samples (*n* = 4), these findings should be considered exploratory.

The immunomodulatory effect exerted by EVs on cultured CeD gut mucosa was further confirmed by the analysis of IFN-γ/IL-10 ratio (Figure 4C) which showed a numerical reduction in the IFN-γ/IL-10 ratio in the presence of MSC-cEVs, although this difference did not reach statistical significance (*p* = 0.125).

## 3. Discussion

CeD, the most frequent chronic enteropathy worldwide [24], is a privileged condition since both the external trigger (gluten) and the autoantigen (the enzyme tTG) have been identified, together with the HLA haplotypes conferring genetic susceptibility [1]. Moreover, the fine pathogenic mechanisms responsible for intestinal lesions have been dissected, with antigen-specific IFN-γ- and IL-21-producing T cells playing a pivotal role in gut mucosal inflammation [25,26,27,28]. Notably, tTG also operates a specific deamidation of gliadin peptides, giving rise to immunodominant antigens [29]. The only available therapy is still based on a strict and lifelong GFD, as originally proposed by the Dutch pediatrician Dr. Willem-Karel Dicke, which is undoubtedly efficacious but constrains patients to dietary restriction, potentially giving rise to chronic psychological distress [2]. MSCs exert broad immunomodulatory effects, particularly following inflammatory activation, mostly by IFN-γ [30], and have been shown to influence immune-cell responses, regulatory pathways, and tissue repair [30,31]. Our group previously demonstrated that bone marrow-derived MSCs can modulate the response of gliadin-specific TCLs [18]. However, the clinical translation of cell-based MSC therapy may be limited by issues related to cell administration, scalability, and safety, particularly in a highly prevalent condition such as CeD [10,24].

EVs comprise a heterogeneous group of membrane-bound particles released by cells and are currently described and classified according to multiple physical, molecular, and biological characteristics rather than by a simple distinction between exosomes and microvesicles [32,33]. In the present study, we use the term “EVs” as a broad designation for the vesicular preparations investigated, in accordance with current recommendations [32,33].

EVs can modulate recipient-cell functions through the transfer of bioactive cargo [11,34]. In this context, MSC- EVs have attracted interest as a potential cell-free approach for immunomodulation, with possible advantages over cell-based MSC therapies in terms of their development and therapeutic application [11,14,34].

EVs have shown immunomodulatory and regenerative properties in several experimental models, including inflammatory and autoimmune diseases [35,36,37,38,39,40,41,42,43]. Notably, MSC-EV preparations have also shown therapeutic potential in preclinical models and clinical studies of refractory acute graft-versus-host disease [44].

The lack of a CeD animal model [5], prompted us to use two widely accepted models, i.e., in vitro short-term TCLs that are reactive to gliadin and ex vivo organ cultures of treated celiac gut mucosa [15,16] that reproduce the main pathogenic mechanisms leading to enteropathy. We found that MSC-EVs display a potent and dose-dependent inhibitory effect on IFN-γ release by short-term TCLs under antigen stimulation. Remarkably, this effect was highly specific, as EVs obtained from fibroblast were not effective, and it did not depend on the primed or unprimed state of MSCs. Although the mechanism through which MSC-EVs exert the suppression of T-cell response to gliadin is not yet elucidated, our findings do not support a major effect of EVs on the surface immunophenotype of antigen-presenting B cells used in the functional assay. More specifically, the surface expression of HLA-DQ/DR, and of costimulatory molecules, CD80/CD83, on B cells pulsed with gliadin plus EVs, compared to cells pulsed with gliadin alone, remained unchanged. However, these findings do not exclude potential effects of MSC-EVs on other aspects of antigen processing or presentation that are not reflected by changes in the expression of these surface markers.

We next investigated the effects of EVs in an ex vivo organ-culture model using duodenal specimens from treated CeD patients. Although the mucosa of treated patients may appear histologically normal following a strict GFD, it remains immunologically responsive to gliadin exposure [16,17,21]. In this tissue-based model, EV treatment reduced the migration of CD3+ T cells into the epithelium and the number of CD25+ cells in the lamina propria following gliadin stimulation, indicating that EVs can influence cellular responses within the intestinal mucosal environment.

The different effects of NHDF-EVs observed across the two experimental systems are of particular interest. The gliadin-specific short-term TCLs assay represents an isolated and focused experimental system that primarily assesses the Th1-associated response of gluten-reactive T cells, with antigen-induced IFN-γ production as the main readout. In this setting, MSC-EVs reduced gliadin-induced IFN-γ secretion, whereas neither unprimed nor IFN-γ-primed NHDF-EVs significantly affected this readout. Thus, within this specific experimental model and for the IFN-γ readout, the inhibitory effect was observed with MSC-derived EVs but not with NHDF- EVs.

In contrast, the ex vivo organ-culture model preserves tissue architecture and cellular interactions within the duodenal mucosa, including epithelial cells, stromal cells, lamina propria cells, and resident immune populations. Consequently, EVs may influence several interconnected processes, including inflammatory-cell recruitment, immune-cell activation, and tissue–immune cell interactions, in addition to suppressing the intrinsic capacity of gliadin-reactive T cells to produce IFN-γ. In this tissue-based model, MSC-cEVs were associated with a reduction in CD3+ intraepithelial cells, whereas NHDF-cEVs showed a similar trend that did not reach statistical significance. For CD25+ lamina propria cells, a statistically significant reduction was observed with NHDF-cEVs, whereas the reduction observed with MSC-cEVs did not reach statistical significance In the subgroup of organ-culture samples analyzed for IFN-γ, both EV preparations were associated with lower IFN-γ levels, although these differences did not reach statistical significance and should therefore be considered exploratory. Thus, the effects observed in the organ-culture model cannot be considered specific to MSC-EVs.

Previous studies have reported immunomodulatory and tissue-repair properties of NHDF-EVs [45,46], which may contribute to their effects in the more complex organ-culture environment.

Although the molecular cargo of NHDF-EVs may differ from that of MSC-EVs, NHDF-EVs can also carry bioactive proteins, lipids, and nucleic acids capable of modulating recipient-cell functions [45,46]. In the multicellular organ-culture environment, these EVs may therefore act on epithelial, stromal, and immune cells and indirectly influence inflammatory-cell recruitment, cellular activation, or tissue–immune cell interactions. This may provide a possible explanation for the overlapping effects observed with NHDF-EVs and MSC-EVs in the organ-culture model, despite the absence of a significant effect of NHDF-EVs on IFN-γ secretion in the isolated T-cell assay.

These findings indicate that the biological effects of EVs are both context- and readout- dependent, and that the absence of an effect in the short-term T-cell assay does not necessarily indicate a lack of biological activity in a more complex tissue environment.

IL-10 is a key regulatory cytokine involved in limiting excessive immune activation and has been implicated in the control of intestinal inflammation in the CeD gut mucosa [22,23,47,48]. In this context, the increase in IL-10 observed in EV-treated organ cultures may be consistent with the induction or reinforcement of regulatory mechanisms within the gliadin-challenged intestinal mucosa. In parallel, the reduction in IFN-γ secretion observed in the presence of EVs may suggest an attenuation of IFN-γ-associated immune activation. Although these findings do not establish a direct anti-inflammatory mechanism or identify the cellular and molecular pathways responsible for these effects, the concomitant modulation of IL-10 and IFN-γ is consistent with the possibility that EVs influence selected regulatory and inflammatory components of the immune response to gliadin. Further studies assessing additional inflammatory and regulatory mediators, as well as the cellular targets of EVs within the intestinal mucosa, will be necessary to determine the extent and mechanisms of their immunomodulatory activity.

Several limitations should be considered when interpreting the overall findings of this work. First, although the reduction in IFN-γ secretion observed in the gliadin-specific T-cell assay is consistent with an immunomodulatory effect of MSC-EVs, we cannot exclude the possibility that changes in T-cell viability, proliferation, or activation may have contributed to the observed reduction, as these parameters were not directly assessed in the present study. Further experiments incorporating appropriate viability, proliferation, and activation assays will therefore be required to clarify the mechanisms underlying the reduction in IFN-γ secretion. The relatively small sample size represents an important limitation of this study and may have reduced the statistical power to detect differences and increased the uncertainty of the statistical estimates. The results should be interpreted with caution, particularly when considering multiple comparisons, and the observed effects should be regarded as exploratory.

In addition, for the functional experiments, MSC-derived EV preparations from individual donors were pooled before use. Although this approach was adopted to reduce interindividual variability in EV potency, it prevented us from assessing donor-to-donor variability and determining whether the observed effects were consistently reproduced across individual EV donor preparations. Future studies using independently tested donor-derived EV preparations will be important to assess the reproducibility and robustness of the observed effects.

Larger, adequately powered studies will be necessary to confirm these findings and to establish their reproducibility and biological relevance. The relatively limited number of donors and patients included in the study did not allow us to adequately assess whether age may influence EV potency or the responsiveness of intestinal immune cells. We have therefore avoided drawing conclusions regarding the influence of age and consider this an additional limitation of the present study. Future studies including a larger number of donors and patients, with appropriate age stratification, will be necessary to determine whether donor or patient age influences EV potency or cellular responsiveness.

With regard to EV characterization, transmission electron microscopy (TEM) analysis of the EV isolates is not available in the present study. Although TEM is recommended as a complementary approach to provide morphological information on EV preparations, it is not considered an obligatory characterization method according to the current International Society for Extracellular Vesicles (ISEV) guidelines. In the present study, EV preparations were characterized by protein quantification, nanoparticle tracking analysis, and multiplex analysis of surface markers; however, TEM was not included in the original experimental workflow. The absence of TEM-based morphological characterization should nevertheless be considered a limitation of the present study, and future studies should include TEM or another suitable imaging-based approach as a complementary characterization of EV morphology, as previously described [49].

Taken together, these findings indicate that EV-mediated effects are influenced by the parental cell source, the biological complexity of the target tissue, and the specific experimental readout. The short-term gliadin-specific TCLs provides a focused assessment of the capacity of EVs to modulate antigen-induced Th1-associated cytokine production, particularly IFN-γ secretion, whereas the ex vivo organ-culture allows the evaluation of EV effects within the multicellular and structural context of the celiac intestinal mucosa. In the former model, the reduction in IFN-γ secretion was observed with MSC-EVs but not with NHDF-EVs, whereas both EV preparations were associated with modulation of cellular responses and IFN-γ secretion in organ cultures. The additional increase in IL-10 and reduction in the IFN-γ/IL-10 ratio observed with MSC-EVs may indicate a broader modulation of the inflammatory/regulatory balance, although the underlying mechanisms remain to be established.

Overall, our findings suggest that MSC-EVs can modulate selected components of the immune response to gliadin, including antigen-induced IFN-γ production by gliadin-reactive T cells, and provide a rationale for further preclinical investigation of EV-mediated immunomodulation in CeD. Importantly, the effects observed in the organ-culture model were not exclusive to MSC-EVs, as NHDF-EVs also modulated cellular responses and IFN-γ secretion. The use of EVs may offer several advantages over their cellular MSC counterparts, including potentially greater stability and more flexible manufacturing, storage, and delivery [11,47,48]. Nevertheless, EV potency may vary according to the parental cell source, the physiological state of the producing cells, and the characteristics of the target cells and tissues [50]. Several challenges must therefore be addressed before MSC-EV-based approaches can be considered for further translational development, including scalable manufacturing, product characterization and identity, potency assays, pharmacokinetics, biodistribution, safety, and definition of their mechanisms of action [51,52]. Due to the limited number of samples included in the present study, these findings should be considered preliminary and do not establish therapeutic efficacy, safety, pharmacokinetic properties, or effective delivery in vivo. Further studies are warranted to identify the specific EV components involved in the modulation of gliadin-induced immune responses, clarify their effects on antigen processing and presentation, and determine whether these effects can be reproduced and translated into appropriate preclinical in vivo models.

## 4. Materials and Methods

### 4.1. Isolation, Expansion and Characterization of Human Mesenchymal Stromal Cells

Bone marrow aspirates (50 mL) were obtained from healthy adult donors after written informed consent, in accordance with the protocol approved by the local Ethics Committee (Comitato Etico per la Sperimentazione Clinica delle Province di Verona e Rovigo, N. Prog. 1828, 12 May 2010: “Institution of cell and tissue collection for biomedical research in Onco-Haematology”). MSC-derived EVs were generated from two independent donor pools, comprising six donors in the first pool and three additional donors in the second pool, for a total of nine biological donors and two independent EV preparations), and were processed as previously described by our group [17]. Briefly, the samples were cultured in tissue flasks at cell concentration of 1 × 10^5^ nucleated cells/cm^2^ in Dulbecco’s Modified Eagle Medium, supplemented with 10% heat-inactivated fetal bovine serum, 2% L-glutamine and 1% penicillin/streptomycin (all by Merck Life Science S.r.l., Milan, Italy.). After 72 h incubation at 37 °C in a 5% CO_2_ humidified atmosphere, non-adherent cells were removed, and the medium was replaced twice a week. Adherent cells were seeded into a new culture flask when reaching 90% of confluence and used for experiments at passages 3 to 7, when their characterization was carried out. Specifically, MSC batches had to achieve the following identification criteria: spindle-shaped morphology as assessed by optical microscopy, immune phenotype showing the expression of CD73, CD90 and CD105 surface molecules (>95%), and the absence of CD14, CD31, CD34, and CD45 as well as HLA-ABC and HLA-DR markers [53]. In addition, the differentiation ability towards mesodermal cell lineage was assessed by culturing the cells in the presence of adipogenic and osteogenic differentiation media [53]. The final batches of MSCs from each bone marrow blood aspirate were separated into those unstimulated that were cryopreserved until use, and those stimulated (primed) that were further cultured for 48 h in the presence of human recombinant interferon (IFN)-γ (25 ng/mL/daily; R&D Systems, Minneapolis, MN, USA) to unravel whether EVs from primed MSCs display stronger immune modulatory activity [19].

### 4.2. Purification and Characterization of Extracellular Vesicles

Purification and characterization of MSC-EVs was carried out as previously described [54]. Briefly, MSCs were first washed with phosphate-buffered saline and then with 10% EV-depleted fetal bovine serum.

Supernatants from both primed and unprimed cell cultures were collected and subjected to sequential centrifugation steps: 10 min at room temperature at 400× *g*, followed by 30 min at 4 °C at 2000× *g* to remove cells, cellular debris, and apoptotic bodies. The supernatants were ultracentrifuged twice for 90 min at 4 °C at 100,000× *g* to collect EVs (Optima XPN-80 Ultracentrifuge, Beckman Coulter, Brea, CA, USA). Purified EVs were resuspended in phosphate-buffered salinealiquoted in final volumes ranging from 50 to 100 µL for each EV preparation, and used for characterization assays or stored at −80 °C until use.

EV characterization was performed according to the criteria adopted by the International Society for Extracellular Vesicles (ISEV) [55] and further optimized by our group [56]. Protein concentration was determined using the bicinchoninic acid (BCA) assay (Thermo Fisher Scientific, Waltham, MA, USA). Characterization of EV size and concentration was performed by Nanoparticle Tracking Analysis (NTA) on a NanoSight NS300 (Malvern Panalytical Ltd., Malvern, Worcestershire, United Kingdom (UK) equipped with a 532 nm laser module. Prior to analysis, samples were vortexed and diluted (1:400–1:1000) to fall within the recommended operational range of the instrument. Video recordings (three 60 s replicates per sample) were collected at a controlled flow rate (syringe pump speed 20) and analyzed with NTA 3.4 software. The resulting size distribution graphs and concentration values, adjusted for dilution, are presented in Supplementary Material (Supplementary Figure S2, and Supplementary Table S1).

Protein concentrations determined by BCA were 0.49 µg/µL for MSC-cEV (EV batches from unstimulated MSCs); 0.25 µg/µL for MSC-pEV (EV batches from primed MSCs); 0.44 µg/µL for NHDF-cEV (EV batches from unstimulated fibroblasts), and 0.224 µg/µL for NHDF-pEV (Ev batches from primed fibroblasts).

In addition, surface-marker expression was analyzed using the MACSPlex Exosome Kit (Miltenyi Biotec B.V. & Co. KG, Bergisch Gladbach, Germany), a multiplex bead-based platform enabling the detection of 37 surface epitopes (IgG1 CTRL, CD1c, CD2, CD3, CD4, CD8, CD9, CD11c, CD14, CD19, CD20, CD24, CD25, CD29, CD31, CD40, CD44, CD45, CD49e, CD69, CD86, CD105, CD133, CD142, CD146, CD209, CD326, MCSP, ROR1, REA CTRL, CD41b, CD42a, CD56, CD62P, HLA-ABC, HLA-DRDPDQ, and SSEA-4) by flow cytometry using a FACSCanto II system (BD Biosciences, San Jose, CA, USA), with 10 μg of each EV preparation, as previously described by our group [19].

For the in vitro and ex vivo functional experiments, the two independently generated MSC-EV preparations were pooled into a single preparation before use. Thus, although MSC-EVs originated from nine biological donors and were generated as two independent EV preparations, the two preparations were not tested independently in the functional experiments. The pooled MSC-EV preparation was therefore the experimental EV material used in the functional assays.

EV batches derived from Primary Normal Human Dermal Fibroblasts (EV-NHDFs), were obtained from a single commercial NHDF preparation (Lonza, Basel, Switzerland). EVs were generated from this single NHDF source under resting or IFN-γ-primed conditions and processed using the same isolation and characterization procedure. Thus, the NHDF-EV experiments were performed using EVs derived from one biological source and did not include independent biological donor replicates.

### 4.3. Production of the Gliadin Antigen

Purified gliadin was enzymatically digested with pepsin and trypsin as previously described [57]. Briefly, wheat gliadin (100 mg; Sigma-Aldrich; Saint Louis, MO, USA) was diluted in 0.1N HCl (10 mL) and incubated with 500 µg pepsin (Sigma-Aldrich) for 2 h at 37 °C, followed by a 2-h incubation with 500 µg trypsin (Sigma-Aldrich) at pH 7.8. Aliquot of peptic-tryptic gliadin preparation were deamidated by 4-h tissue-transglutaminase (tTG) (Sigma-Aldrich) treatment in 0.125 mol/L Tris-HCl, pH 8.5, (ratio tTG/gliadin1:10), as previously described [16]. Finally, antigenic aliquots were dried and stored at −80 °C until use. The tTG-deamidated peptic-tryptic digested gliadin is indicated as gliadin hereafter.

### 4.4. Coeliac Disease Patients

Overall, 13 patients with biopsy-proven CeD and whose demographic and clinical features are presented in Table 1 were enrolled in this study.

Of them, five were paediatric patients (mean age: 7.5 years; range: 2.2–15.3) and recruited at the Department of Translational Medical Sciences, Section of Paediatrics, University of Naples Federico II (Italy). The remaining eight were adult patients (median age 40 years, range: 17–55) enrolled at the Gastroenterology Unit, San G. Moscati Hospital, Avellino (Italy). Perendoscopic duodenal biopsies taken from five treated children and one adult patient with active CeD were used to generate short-term gliadin-specific TCLs (TCLs), whereas those from seven adult patients on a GFD for at least two years were used for organ culture experiments. The study was approved by the Ethics Committee of the University of Naples “Federico II” (protocol No. CE183/19, 4 July 2019), and by the Ethics Committee of Campania Nord (protocol No. CECN-819, 21 March 2018, and protocol No. CECN-2035, 22 March 2023). Informed consent was obtained from all participants, or from parents/legal guardians in the case of children under 14 years of age, after a full explanation of the study objectives was given.

### 4.5. Generation of Intestinal Gliadin-Specific T Cell Lines

The intestinal T cells were isolated from endoscopic duodenal specimens of CeD patients, as previously described and fine-tuned by our group [20]. Briefly, after harvesting, biopsies were digested with 1.5 mg/mL Collagenase A (Roche; Basel, Switzerland) and incubated for 1 h and 30 min at 37 °C and 5% CO2. Cell viability and recovery rates were assessed using trypan blue dye exclusion, and living intestinal cells were plated at density of 5 × 10^5^ cells/well in the presence of 1.5 × 10^6^ irradiated (35Gy = 3500 rad) autologous peripheral blood mononuclear cells (PBMCs) isolated from patients ‘own peripheral blood samples using a Ficoll-Paque gradient density, in a total volume of 1.5 mL of complete culture medium (X-vivo supplemented with 5% human serum and penicillin and streptomycin antibiotics) and gliadin at 50 μg/mL final concentration. The day after and then every three days, cells were fed with 500 μL of complete medium and interleukin (IL)-2 and IL-15 as growth factors, added at concentrations of 20 U/mL and 10 ng/mL, respectively (both from R&D System; Minneapolis, MN, USA). The short-term TCLs underwent two cycles of stimulation with gliadin, on days 7–9 and on days 21–24 after the set-up. Thereafter, T cells were maintained in culture by cyclic stimulation with phytohemagglutinin.

### 4.6. Generation of Immortalized B Cells and Immunophenotype Analysis

The B-lymphoblastoid cell lines were established from PBMCs of five HLA-DQ2.5 CeD patients by infection with a culture medium of Marmoset cells, previously titrated for Epstein-Barr virus virulence, in the presence of cyclosporine A at 0.5 μg/mL. Then, the B-lymphoblastoid cell lines were pre-pulsed for 2 h with 50 μg/mL gliadin and then incubated with: MSC-cEV, MSC-pEV, NHDF-cEV and NHDF-pEV (EV batches from primed fibroblasts) at 8 µg/mL for 48 h. Finally, the cells were stained with anti-HLA-DQ-APC (cat.No: 130-104-546, Miltenyi Biotec B.V. & Co. KG, 51429 Bergisch Gladbach, Germany, clone: REA303),anti-HLA-DR-PerCP (cat.No: 2138140 Sony Biotecnology Inc., San Jose, CA, USA (95112); clone: L243), anti-CD86-AF750 (cat.No: 33435X, BD Pharmingen, San Diego, CA, USA (92121), clone: II/41), anti-CD83-FITC (cat.No:2126650,Sony Biotecnology Inc., San Jose, CA, USA (95112), clone: HB15e) monoclonal antibodies and the mean fluorescence intensity (MFI) was measured by FACS Canto instrument (BD Biosciences, 155 McCarthy Boulevard, Milpitas, CA 95035, USA) using the BD FACSDiva Software, version 8.0 (BD Biosciences, Milan, Italy).

### 4.7. Evaluation of MSC-EV Immunosuppressive Effect on Gliadin-Specific T Cells

Autologous or HLA-matched allogeneic immortalized B-cells (6 × 10^4^/well) were pre-incubated with gliadin (50 μg/mL) for two hours at 37 °C in 96-well round bottom plates. Thereafter, gliadin-specific T cells were incubated for 1 h with the EV preparations, as follows: MSC-cEVs; MSC-pEVs; NHDF-cEVs, NHDF-pEVs used at 2, 4, and 8 μg/mL concentration. After incubations, T cells were added to B cells pre-pulsed with gliadin in a final volume of 200 μL of complete medium and were incubated for additional 48 h. After that, 50 μL of each cell supernatant was collected to assess INF-γ concentration by a standard sandwich ELISA developed in house. Both functional T-cell assays and IFN-γ ELISA measurements were performed in technical duplicate.

### 4.8. ELISA Assay

A sandwich ELISA was performed over 3 days to quantify IFN-γ production in the culture supernatants of gliadin-specific st-TCLs (*n* = 6). Briefly, on day 1, 50 μL/well of purified anti-human IFN-γ capture antibody (Mabtech AB, 131 52 Nacka Strand, Sweden; cod. 3420-3-1000; final concentration 1 μg/mL), diluted in 0.1 M NaHCO_3_ coating buffer, was added to an EIA/RIA 96-well plate (Cat. No. 3369, Costar) and incubated overnight at 4 °C. On day 2, the plate was washed three times with PBS containing 0.05% Tween-20 (Sigma-Aldrich, St. Louis, MO 63103, USA) and blocked with 200 μL/well of PBS containing 2% BSA for 2 h at room temperature. After three additional washes, 50 μL/well of culture supernatants were added. Recombinant IFN-γ (BD Biosciences, San Jose, CA, USA, cod. 554617) was included on each plate as a standard and serially diluted 1:2 in blocking buffer. The plate was then incubated overnight at 4 °C. On day 3, following three washes, 50 μL/well of biotinylated anti-human IFN-γ detection antibody (Mabtech AB, 131 52 Nacka Strand, Sweden; cod. 3420-6-250; final concentration 1 μg/mL), diluted in blocking buffer, was added and incubated for 1 h at room temperature. After three further washes, 100 μL/well of peroxidase-conjugated streptavidin (SAv-HRP; BD Pharmingen, San Diego, CA 92121, USA), diluted 1:5000 in blocking buffer, was added and incubated for 45 min at RT. The plate was subsequently washed and 100 μL/well of TMB substrate (Sigma-Aldrich St. Louis, MO 63103, USA), prepared in citrate buffer (3.15 g citric acid monohydrate in 500 mL water, pH 4.2; TMB dilution 1:20), was added. Following incubation for 3–5 min in the dark at RT, the reaction was stopped by adding 50 μL/well of 1 NH_2_SO_4_. Absorbance was measured at 450 nm using a Multiskan FC spectrophotometer (Thermo Scientific, Waltham, MA 02451, USA). All samples and standards were analyzed in technical duplicate, and the mean absorbance of the duplicate wells was used for subsequent analyses.

### 4.9. Organ Culture and Immunohistochemistry

Organ cultures of duodenal biopsies obtained from seven adult treated CeD patients were performed as previously reported [17]. Briefly, mucosal specimens were cultured in the presence of medium alone, gliadin (500 μg/mL), gliadin plus MSCs-cEVs and plus NHDFs-cEVs at concentration of 20 μg/mL. After 24 h, the biopsy was embedded in optimal cutting temperature compound (Tissue-Tek; Sakura Finetek Italy S.r.l., 30172 Venice, Italy), and then 5-µm cryostat sections were prepared for immunohistochemical analysis. Lamina propria mononuclear CD25+ cells and CD3+ intaepithelial lymphocytes (IELs) were detected in acetone-fixed serial sections incubated with anti-CD25 (Cat.347640, Clone2A3, 1:25, BD Pharmingen, San Diego, CA 92121, USA) and anti-CD3 (Cat.M0835, Clone UCHT1, 1:200, Agilent Dako, Glostrup, 2600, Denmark) monoclonal antibodies, followed by a goat anti-mouse Alexa fluor 568 (Cat.A11031) and a goat anti-mouse Alexa fluor 488 (Cat.A21121) (both at 1:500 dilution, Molecular Probes, Thermo Fisher Scientific, Eugene, OR 97402, USA), respectively. Finally, sections were counterstained with ToPro-3 (Molecular Probes, Thermo Fisher Scientific, Eugene, OR 97402, USA) and analyzed by two blind observers by using a confocal microscope (Leica TCS SP8 confocal microscope, Leica Microsystems, Wetzlar, 35578, Germany). The number of CD25+ cells was counted within a total area of 1 mm^2^ of lamina propria, whereas the density of CD3+cells in the intraepithelial compartment was evaluated per millimeter of epithelium.

### 4.10. IL-10 and IFN-γ ELISA in Organ Culture Supernatants

After 24 h, the biopsy culture medium was collected and stored at −80 °C. For IFN-γ and IL-10 evaluation, 100 µL of each culture supernatant was assayed in technical triplicate by sandwich ELISA, following the procedure described in Section 4.8. For IFN-γ, capture and detection antibodies were purchased by eBioscience, Thermo Fisher Scientific, San Diego, CA 92121, USA (Cod.14-7318-85 and Cod. 13-7319-81, respectively), human recombinant IFN-γ standard protein was from PeproTech, Cranbury, NJ 08512, USA (Cod. 300-02-20UG) and the assay sensitivity was 15.6 pg/mL. For IL-10 detection, antibody pair was purchased from Mabtech AB, 131 52 Nacka Strand, Sweden (Capture Ab Cod. 3430-3-250, Biotinylated Ab Cod. 3430-6-250), purified standard protein was from Thermo Fisher Scientific, Waltham, MA 02451, USA (Cod. 29-8109-65) and assay sensitivity was 9 pg/mL.

### 4.11. Statistical Analysis

The INF-γ and IL-10 levels in supernatants were expressed as pg/mL and data were shown as the arithmetic median and 25th–75th percentiles. The mean fluorescence intensity (MFI) expression of HLA-DQ/DR, CD83/CD86 on B cells and the immunohistochemical data on CD3 and CD25 positive cells were presented as median and 25th–75th percentiles. Given the small sample size and the non-normal distribution of the data, a Wilcoxon signed-rank test for paired samples was used to compare the response to gliadin in the presence and absence of MSC/NHDF-EVs at the different concentrations tested in both in vitro and ex vivo experiments. A two-sided *p*-value < 0.05 was considered statistically significant. Statistical analyses were performed using GraphPad Prism Software (Version 8, San Diego, CA, USA) were used for computation. No artificial intelligence generated content tool was used in this study.

## Figures and Tables

**Table 1:** Clinical features of CeD patients enrolled.

Subjects	Gender	Age (Years)	Anti-tTG IgA/EMA	Histology *
**Subjects enrolled for generation of gliadin-specific TCLs**				
**#1**	Female	15.3	145 (U/mL)	Type 3
**#2**	Female	2.2	100 (U/mL)	Type 3
**#3**	Female	34	Not available	Type 3
**#4**	Female	2.6	41 (U/mL)	Type 1
**#5**	Female	12.2	48.9 (U/mL)	Type 0
**#6**	Female	5.3	100 (U/mL)	Type 3
**Subjects enrolled for organ culture experiments**				
**#7**	Female	55	Negative	Type 1
**#8**	Female	17	Negative	Type 0
**#9**	Male	40	nd	Type 2
**#10**	Female	36	nd	Type 1
**#11**	Female	54	Negative	Type 0
**#12**	Female	27	Negative	Type 2
**#13**	Female	49	Negative	Type 1

* Grading of mucosal lesions are presented according to the Marsh score, as follows: type 0: no infiltration; type 1: infiltrative; type 2: hyperplastic; type 3: destructive, by Marsh MN. EMA: anti-endomysial antibody; tTG: tissue transglutaminase.

## Data Availability

The data supporting the findings of this study are available from the corresponding author upon reasonable request. The data are not publicly available due to ethical and privacy restrictions related to the use of human biological samples and patient-derived data.

## Supplementary Materials

The following supporting information can be downloaded at: https://www.mdpi.com/article/10.3390/ijms27209249/s1.

## Abbreviations

The following abbreviation are used in this manuscript:

CeD	Celiac disease
EVs	Extracellular vesicles
GFD	Gluten-free diet
IFN-γ	Interferon-γ
IL	Interleukin
MSC	Mesenchymal stromal cell
NHDF	Normal non-celiac human dermal fibroblast
TCL	T-cell line
tTG	Tissue transglutaminase
cEVs	EVs from control cells
pEVs	EVs from primed cells

## References

[1] Abadie V., Han A.S., Jabri B., Sollid L.M. (2024). New Insights on Genes, Gluten, and Immunopathogenesis of Celiac Disease. Gastroenterology.

[2] Lee A.R., Ng D.L., Diamond B., Ciaccio E.J., Green P.H.R. (2012). Living with Coeliac Disease: Survey Results from the U.S.A. J. Hum. Nutr. Diet..

[3] Wiech P., Chmiel Z., Bazalinski D., Sałacińska I., Bartosiewicz A., Mazur A., Korczowski B., Binkowska-Bury M., Dąbrowski M. (2018). The Relationship between Body Composition and a Gluten-Free Diet in Children with Celiac Disease. Nutrients.

[4] Klonarakis M., Andrews C.N., Raman M., Panaccione R., Ma C. (2022). Review Article: Therapeutic Targets for the Pharmacologic Management of Coeliac Disease—The Future beyond a Gluten-Free Diet. Aliment. Pharmacol. Ther..

[5] Levescot A., Cerf-Bensussan N. (2024). Loss of Tolerance to Dietary Proteins: From Mouse Models to Human Model Diseases. Immunol. Rev..

[6] Regmi S., Pathak S., Kim J.O., Yong C.S., Jeong J.-H. (2019). Mesenchymal Stem Cell Therapy for the Treatment of Inflammatory Diseases: Challenges, Opportunities, and Future Perspectives. Eur. J. Cell Biol..

[7] Niu J.W., Li Y., Xu C., Sheng H., Tian C., Ning H., Hu J., Chen J., Li B., Wang J. (2024). Human Umbilical Cord-Derived Mesenchymal Stromal Cells for the Treatment of Steroid-Refractory Grades III–IV Acute Graft-versus-Host Disease with Long-Term Follow-Up. Front. Immunol..

[8] Ciccocioppo R., Russo M.L., Bernardo M.E., Biagi F., Catenacci L., Avanzini M.A., Alvisi C., Vanoli A., Manca R., Luinetti O. (2012). Mesenchymal Stromal Cell Infusions as Rescue Therapy for Corticosteroid-Refractory Adult Autoimmune Enteropathy. Mayo Clin. Proc..

[9] Ciccocioppo R., Gallia A., Avanzini M.A., Betti E., Picone C., Vanoli A., Paganini C., Biagi F., Maccario R., Corazza G.R. (2016). A Refractory Celiac Patient Successfully Treated with Mesenchymal Stem Cell Infusions. Mayo Clin. Proc..

[10] Musial-Wysocka A., Kot M., Majka M. (2019). The Pros and Cons of Mesenchymal Stem Cell-Based Therapies. Cell Transplant..

[11] Mokarizadeha A., Delirezh N., Morshedi A., Mosayebi G., Farshid A.-A., Mardani K. (2012). Microvesicles Derived from Mesenchymal Stem Cells: Potent Organelles for Induction of Tolerogenic Signaling. Immunol. Lett..

[12] Yates A.G., Pink R.C., Erdbrügger U., Siljander P.R., Dellar E.R., Pantazi P., Akbar N., Cooke W.R., Vatish M., Dias-Neto E. (2022). In Sickness and in Health: The Functional Role of Extracellular Vesicles in Physiology and Pathology In Vivo. Part I: Health and Normal Physiology. J. Extracell. Vesicles.

[13] Yates A.G., Pink R.C., Erdbrügger U., Siljander P.R., Dellar E.R., Pantazi P., Akbar N., Cooke W.R., Vatish M., Dias-Neto E. (2022). In Sickness and in Health: The Functional Role of Extracellular Vesicles in Physiology and Pathology In Vivo. Part II: Pathology. J. Extracell. Vesicles.

[14] Baglio S.R., Pegtel D.M., Baldini N. (2012). Mesenchymal Stem Cell Secreted Vesicles Provide Novel Opportunities in (Stem) Cell-Free Therapy. Front. Physiol..

[15] Camarca A., Anderson R.P., Mamone G., Fierro O., Facchiano A., Costantini S., Zanzi D., Sidney J., Auricchio S., Sette A. (2009). Intestinal T Cell Responses to Gluten Peptides Are Largely Heterogeneous: Implications for a Peptide-Based Therapy in Celiac Disease. J. Immunol..

[16] Gianfrani C., Maglio M., Rotondi Aufiero V., Camarca A., Vocca I., Iaquinto G., Giardullo N., Pogna N., Troncone R., Auricchio S. (2012). Immunogenicity of Monococcum Wheat in Celiac Patients. Am. J. Clin. Nutr..

[17] Gianfrani C., Camarca A., Mazzarella G., Di Stasio L., Giardullo N., Ferranti P., Picariello G., Rotondi Aufiero V., Picascia S., Troncone R. (2015). Extensive In Vitro Gastrointestinal Digestion Markedly Reduces the Immune-Toxicity of *Triticum monococcum* Wheat: Implication for Celiac Disease. Mol. Nutr. Food Res..

[18] Ciccocioppo R., Camarca A., Cangemi G.C., Radano G., Vitale S., Betti E., Ferrari D., Visai L., Strada E., Badulli C. (2014). Tolerogenic Effect of Mesenchymal Stromal Cells on Gliadin-Specific T Lymphocytes in Celiac Disease. Cytotherapy.

[19] Adamo A., Brandi J., Caligola S., Delfino P., Bazzoni R., Carusone R., Cecconi D., Giugno R., Manfredi M., Robotti E. (2019). Extracellular Vesicles Mediate Mesenchymal Stromal Cell-Dependent Regulation of B Cell PI3K-AKT Signaling Pathway and Actin Cytoskeleton. Front. Immunol..

[20] Pisapia L., Picascia S., Farina F., Barba P., Gianfrani C., Del Pozzo G. (2020). Differential Expression of Predisposing HLA-DQ2.5 Alleles in DR5/DR7 Celiac Disease Patients Affects the Pathological Immune Response to Gluten. Sci. Rep..

[21] Mazzarella G., Maglio M., Paparo F., Nardone G., Stefanile R., Greco L., van de Wal Y., Kooy Y., Koning F., Auricchio S. (2003). An Immunodominant DQ8-Restricted Gliadin Peptide Activates Small Intestinal Immune Response in In Vitro Cultured Mucosa from HLA-DQ8-Positive but Not HLA-DQ8-Negative Coeliac Patients. Gut.

[22] Gianfrani C., Levings M.K., Sartirana C., Mazzarella G., Barba G., Zanzi D., Camarca A., Iaquinto G., Giardullo N., Auricchio S. (2006). Gliadin-Specific Type 1 Regulatory T Cells from the Intestinal Mucosa of Treated Celiac Patients Inhibit Pathogenic T Cells. J. Immunol..

[23] Camarca A., Rotondi Aufiero V., Mazzarella G. (2023). Role of Regulatory T Cells and Their Potential Therapeutic Applications in Celiac Disease. Int. J. Mol. Sci..

[24] Lionetti E., Gatti S., Pulvirenti A., Catassi C. (2015). Celiac Disease from a Global Perspective. Best Pract. Res. Clin. Gastroenterol..

[25] Olaussen R.W., Johansen F.E., Lundin K.E., Jahnsen J., Brandtzaeg P., Farstad I.N. (2002). Interferon-Gamma-Secreting T Cells Localize to the Epithelium in Coeliac Disease. Scand. J. Immunol..

[26] Nilsen E.M., Lundin K.E., Krajci P., Scott H., Sollid L.M., Brandtzaeg P. (1995). Gluten-Specific, HLA-DQ-Restricted T Cells from Coeliac Mucosa Produce Cytokines with Th1 or Th0 Profile Dominated by Interferon Gamma. Gut.

[27] Fina D., Sarra M., Caruso R., Blanco G.D.V., Pallone F., MacDonald T.T., Monteleone G. (2008). Interleukin 21 Contributes to the Mucosal T Helper Cell Type 1 Response in Coeliac Disease. Gut.

[28] Bodd M., Ráki M., Tollefsen S., Fallang L.E., Bergseng E., E A Lundin K., Sollid L.M. (2010). HLA-DQ2-Restricted Gluten-Reactive T Cells Produce IL-21 but Not IL-17 or IL-22. Mucosal Immunol..

[29] Molberg O., McAdam S.N., Körner R., Quarsten H., Kristiansen C., Madsen L., Fugger L., Scott H., Norén O., Roepstorff P. (1998). Tissue Transglutaminase Selectively Modifies Gliadin Peptides That Are Recognized by Gut-Derived T Cells in Celiac Disease. Nat. Med..

[30] Silva-Carvalho A.E., Resende Sousa M.R., Alencar-Silva T., Lott Carvalho J., Saldanha-Araujo F. (2019). Mesenchymal Stem Cells Immunomodulation: The Road to IFN-γ Licensing and the Path Ahead. Cytokine Growth Factor Rev..

[31] Giacomini C., Graneli C., Hicks R., Dazzi F. (2023). The Critical Role of Apoptosis in Mesenchymal Stromal Cell Therapeutics and Implications in Homeostasis and Normal Tissue Repair. Cell. Mol. Immunol..

[32] Lötvall J., Hill A.F., Hochberg F., Buzás E.I., Di Vizio D., Gardiner C., Gho Y.S., Kurochkin I.V., Mathivanan S., Quesenberry P. (2014). Minimal Experimental Requirements for Definition of Extracellular Vesicles and Their Functions: A Position Statement from the International Society for Extracellular Vesicles. J. Extracell. Vesicles.

[33] Vaiaki E.M., Falasca M. (2024). Comparative Analysis of the Minimal Information for Studies of Extracellular Vesicles Guidelines: Advancements and Implications for Extracellular Vesicle Research. Semin. Cancer Biol..

[34] Katsuda T., Kosaka N., Takeshita F., Ochiya T. (2013). The Therapeutic Potential of Mesenchymal Stem Cell-Derived Extracellular Vesicles. Proteomics.

[35] Gurung S., Perocheau D., Touramanidou L., Baruteau J. (2021). The Exosome Journey: From Biogenesis to Uptake and Intracellular Signalling. Cell Commun. Signal..

[36] Kim H., Joung Lee M., Bae E.-H., Ryu J.S., Kaur G., Kim H.J., Kim J.Y., Barreda H., Jung S.Y., Choi J.M. (2020). Comprehensive Molecular Profiles of Functionally Effective MSC-Derived Extracellular Vesicles in Immunomodulation. Mol. Ther..

[37] Blazquez R., Sanchez-Margallo F.M., de la Rosa O., Dalemans W., Ãlvarez V., Tarazona R., Gasado J. (2014). Immunomodulatory Potential of Human Adipose Mesenchymal Stem Cells Derived Exosomes on In Vitro Stimulated T Cells. Front. Immunol..

[38] Bruno S., Grange C., Collino F., Deregibus M.C., Cantaluppi V., Biancone L., Tetta C., Camussi G. (2012). Microvesicles Derived from Mesenchymal Stem Cells Enhance Survival in a Lethal Model of Acute Kidney Injury. PLoS ONE.

[39] Li T., Yan Y., Wang B., Qian H., Zhang X., Shen L., Wang M., Zhou Y., Zhu W., Li W. (2012). Exosomes Derived from Human Umbilical Cord Mesenchymal Stem Cells Alleviate Liver Fibrosis. Stem Cells Dev..

[40] Long Q., Upadhya D., Hattiangady B., Kim D.-K., An S.Y., Shuai B., Prockop D.J., Shetty A.K. (2017). Intranasal MSC-Derived A1-Exosomes Ease Inflammation, and Prevent Abnormal Neurogenesis and Memory Dysfunction after Status Epilepticus. Proc. Natl. Acad. Sci. USA.

[41] Hu W., Song X., Yu H., Sun J., Wang H., Zhao Y. (2022). Clinical Translational Potentials of Stem Cell-Derived Extracellular Vesicles in Type 1 Diabetes. Front. Endocrinol..

[42] Maglio M., Florian F., Vecchiet M., Auricchio R., Paparo F., Spadaro R., Zanzi D., Rapacciuolo L., Franzese A., Sblattero D. (2009). Majority of Children with Type 1 Diabetes Produce and Deposit Anti-Tissue Transglutaminase Antibodies in the Small Intestine. Diabetes.

[43] Gianfrani C., Camarca A., Picascia S., Vitale S., Mottola I., Carpinelli M., Maglio M., Gregori S., Franzese A., Auricchio R. (2025). T Cell-Mediated Immunity to Gliadin Is Elicited in the Gut Mucosa of Type 1 Diabetes Patients Only in Presence of Celiac Disease Comorbidity. Diabetes.

[44] Wu P., Wang Z., Sun Y., Cheng Z., Wang M., Wang B. (2025). Extracellular Vesicles: A New Frontier in Diagnosing and Treating Graft-versus-Host Disease after Allogeneic Hematopoietic Cell Transplantation. J. Nanobiotechnol..

[45] Tasnim A., Jacho D., Rabino A., Benalcazar J., Garcia-Mata R., Lapitsky Y., Yildirim-Ayan E. (2025). Immunomodulation through Fibroblast-Derived Extracellular Vesicles (EVs) within 3D Polycaprolactone–Collagen Matrix. Biomimetics.

[46] Ahmadpour F., Mohammadiam Salim M., Reza Esmailinejad M., Razei A., Talebi S., Reza Rasouli H. (2023). Comparison of the Effects of Human Fetal Umbilical Cord-Derived Hyaluronic Acid and Fibroblast-Derived Exosomes on Wound Healing in Rats. Burns.

[47] Passeri L., Andolfi G., Bassi V., Russo F., Giacomini G., Laudisa C., Marrocco I., Cesana L., Di Stefano M., Fanti L. (2023). Tolerogenic IL-10-Engineered Dendritic Cell-Based Therapy to Restore Antigen-Specific Tolerance in T Cell-Mediated Diseases. J. Autoimmun..

[48] Passerini L., Amodio G., Bassi V., Vitale S., Mottola I., Di Stefano M., Fanti L., Sgaramella P., Ziparo C., Furio S. (2024). IL-10-Producing Regulatory Cells Impact on Celiac Disease Evolution. Clin. Immunol..

[49] Dal Collo G., Adamo A., Gatti A., Tamellini E., Bazzoni R., Takam Kamga P., Tecchio C., Quaglia F.M., Krampera M. (2020). Functional Dosing of Mesenchymal Stromal Cell-Derived Extracellular Vesicles for the Prevention of Acute Graft-versus-Host Disease. Stem Cells.

[50] Zhu X., Badawi M., Pomeroy S., Sutaria D.S., Xie Z., Baek A., Jiang J., Elgamal O.A., Mo X., Perle K. (2017). Comprehensive Toxicity and Immunogenicity Studies Reveal Minimal Effects in Mice Following Sustained Dosing of Extracellular Vesicles Derived from HEK293T Cells. J. Extracell. Vesicles.

[51] Silva A.K.A., Morille M., Piffoux M., Arumugam S., Mauduit P., Larghero J., Bianchi A., Aubertin K., Blanc-Brude O., Noël D. (2021). Development of Extracellular Vesicle-Based Medicinal Products: A Position Paper of the Group “Extracellular Vesicle TranslatiOn to clinicaL perspectiVEs—EVOLVE France”. Adv. Drug Deliv. Rev..

[52] Gimona M., Brizzi M.F., Boon Hwa Choo A., Dominici M., Davidson S.M., Grillari J., Hermann D.M., Hill A.F., de Kleijn D., Lai R.C. (2021). Critical Considerations for the Development of Potency Tests for Therapeutic Applications of Mesenchymal Stromal Cell-Derived Small Extracellular Vesicles. Cytotherapy.

[53] Dominici M., Le Blanc K., Mueller I., Slaper-Cortenbach I., Marini F.C., Krause D.S., Deans R.J., Keating A., Prockop D.J., Horwitz E.M. (2006). Minimal Criteria for Defining Multipotent Mesenchymal Stromal Cells. The International Society for Cellular Therapy Position Statement. Cytotherapy.

[54] Théry C., Amigorena S., Raposo G., Clayton A. (2006). Isolation and Characterization of Exosomes from Cell Culture Supernatants and Biological Fluids. Curr. Protoc. Cell Biol..

[55] Théry C., Witwer K.W., Aikawa E., Alcaraz M.J., Anderson J.D., Andriantsitohaina R., Antoniou A., Arab T., Archer F., Atkin-Smith G.K. (2018). Minimal Information for Studies of Extracellular Vesicles 2018 (MISEV2018): A Position Statement of the International Society for Extracellular Vesicles and Update of the MISEV2014 Guidelines. J. Extracell. Vesicles.

[56] Di Trapani M., Bassi G., Midolo M., Gatti A., Kamga P.T., Cassaro A., Carusone R., Adamo A., Krampera M. (2016). Differential and Transferable Modulatory Effects of Mesenchymal Stromal Cell-Derived Extracellular Vesicles on T, B and NK Cell Functions. Sci. Rep..

[57] Gianfrani C., Siciliano R.A., Facchiano A.M., Camarca A., Mazzeo M.F., Costantini S., Salvati V.M., Maurano F., Mazzarella G., Iaquinto G. (2007). Transamidation of Wheat Flour Inhibits the Response to Gliadin of Intestinal T Cells in Celiac Disease. Gastroenterology.

