# Supplementary material for: Immunomodulatory Effects of MSC-Derived ExtracellularVesicles in Celiac Disease
Source: Int J Mol Sci. 2026 Oct 18;27(20):9249. doi: 10.3390/ijms27209249 (PMC13705273; doi:10.3390/ijms27209249)
Supplement: Supplementary file 1 [file ijms-27-09249-s001.zip › ijms-4558495-supplementary.pdf]

Supplementary material

A)

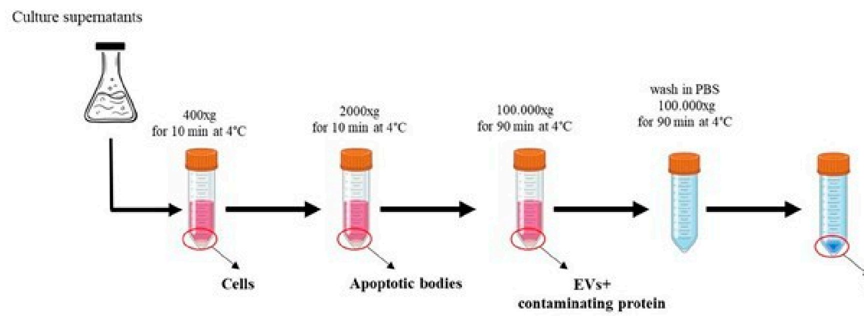

B)

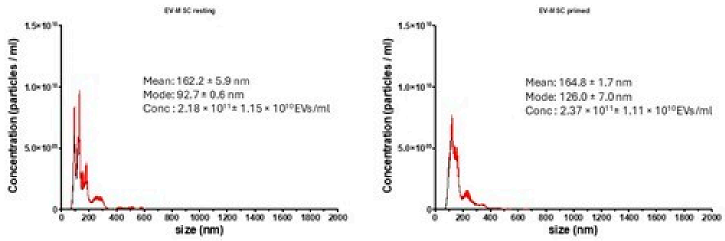

C)

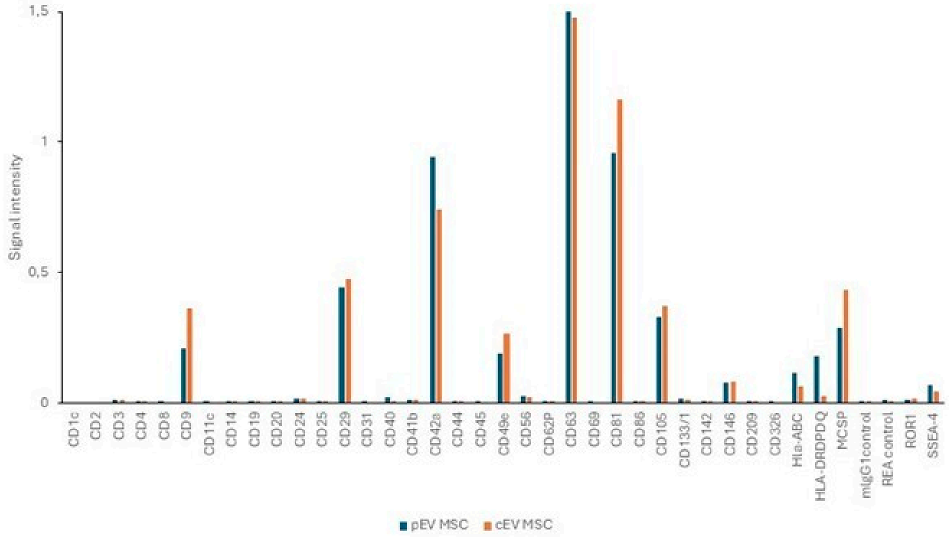

Supplementary Figure S1. Isolation and characterization of EVs from MSC. A: Outline of the differential centrifugation-based protocol used. **B**:Nanoparticle tracking analysis (NTA) of MSC-EV primed and resting. The histograms show the small vescicle quantification and the size distribution. **C**:Mean fluorescence intensity(MFI) of 37 surface epitopes on MCS-EV.

EV preparation	Mean ± SE (nm)	Mode ± SE (nm)	D50 ± SE (nm)	Particle concentration (particles/mL)	Protein (BCA, µg/µL)
**EV-MSC resting**	162.2 ± 5.9	114.4 ± 10.9	133.2 ± 1.1	$2.18 \times 10^{11} \pm 1.15 \times 10^{10}$	0.49
**EV-MSC primed**	164.8 ± 1.7	126.0 ± 7.0	142.5 ± 2.0	$2.37 \times 10^{11} \pm 1.11 \times 10^{10}$	0.25
**EV-NHDF resting**	103.8 ± 2.1	76.6 ± 6.7	88.5 ± 1.7	$7.24 \times 10^{10} \pm 2.42 \times 10^{9}$	0.44
**EV-NHDF primed**	107.5 ± 3.9	77.0 ± 3.6	92.0 ± 1.0	$2.35 \times 10^{11} \pm 2.47 \times 10^{10}$	0.22

Supplementary Table S1. Nanoparticle tracking analysis and protein quantification of extracellular vesicle preparations.
EV size distribution and particle concentration were determined by nanoparticle tracking analysis (NTA; NTA 3.4, Build 3.4.003). Data are reported as mean ± standard error (SE). D50 indicate the particle diameters below which 50% of the measured particles are distributed. Protein concentration was determined by the bicinchoninic acid (BCA) assay and is expressed as µg/µL. EV-MSC resting and primed preparations were obtained from bone-marrow-derived mesenchymal stromal cells (MSCs), whereas EV-NHDF resting and primed preparations were obtained from normal human dermal fibroblasts (NHDFs).

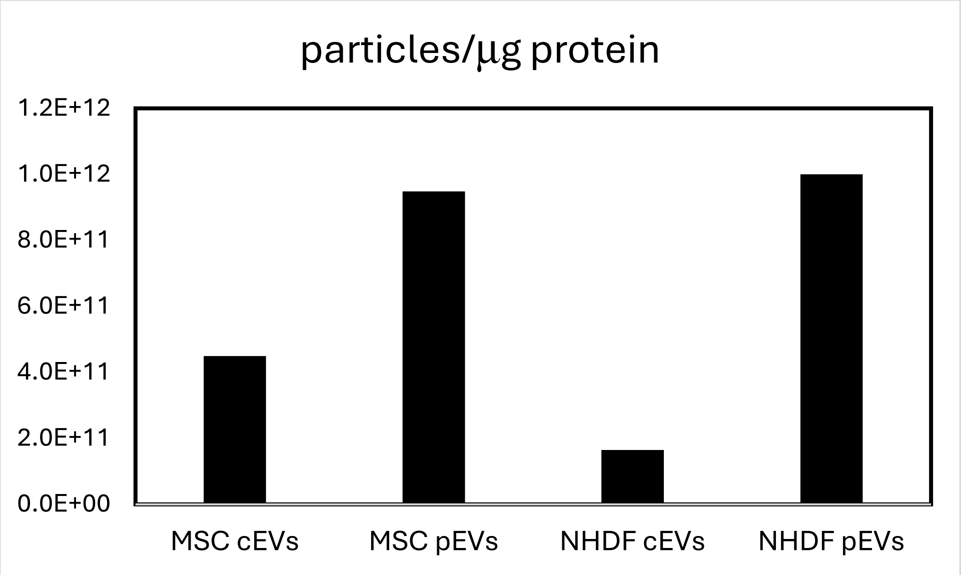

Supplementary Figure S2. Particle-to-protein ratio of EV preparations

Particle-to-protein ratios were calculated by dividing the particle concentration adjusted for dilution determined by nanoparticle tracking analysis (particles/mL) by the protein concentration determined by the bicinchoninic acid (BCA) assay (µg/mL). The ratios were 4.45×10^{11}, 9.48×10^{11}, 1.6×10^{11}, and 1.05×10^{12} particles/µg protein forEV-MSC resting, EV-MSC primed, EV-NHDF resting, and EV-NHDF primed

preparations, respectively. For the EV-NHDF primed preparation, the NTA software indicated that the particle concentration measurement should be interpreted with caution because of noise.

Supplementary Figure S3. EVs reduces activated lamina propria mononuclear cells and T IELs in organ cultured duodenal biopsies

Mucosal sections from a representative coeliac patient cultured in vitro at BASELINE, with GLIA alone or in combination with MSC-cEVs and NHDF-cEVs, are reported. Activated CD25+ cells are shown in red, CD3+ IELs are shown in green and nuclei are counterstained with DAPI (blue). Original magnification:×400.

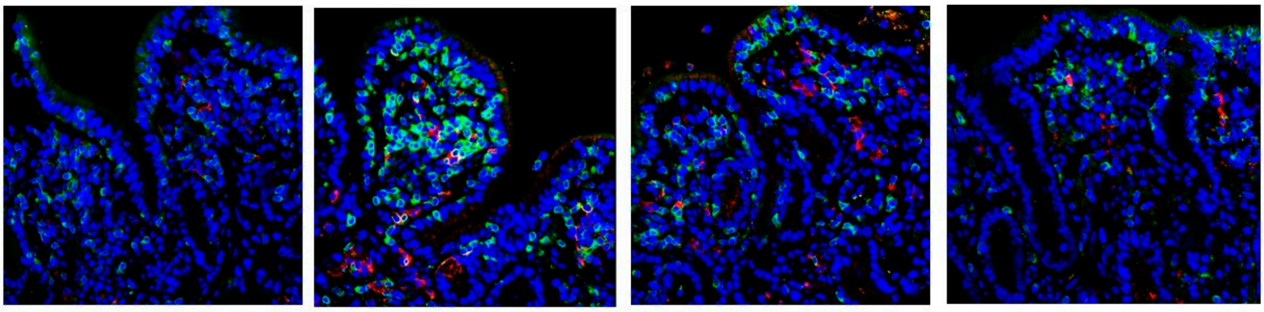

BASELINE GLIA GLIA+MSC cEVs GLIA+NHDF cEVs
